# Initial experience, feasibility and safety of permanent left bundle branch pacing: results from a prospective single-centre study

**DOI:** 10.1007/s12471-021-01648-6

**Published:** 2021-11-26

**Authors:** L. M. Rademakers, J. L. P. M. van den Broek, M. Op ’t Hof, F. A. Bracke

**Affiliations:** Department of Cardiology, Catharina Hospital, Eindhoven, The Netherlands

**Keywords:** Left bundle branch pacing, Physiologic pacing, Cardiac resynchronisation therapy, Bradycardia pacing

## Abstract

**Background:**

Left bundle branch (LBB) pacing is a novel pacing technique which may serve as an alternative to both right ventricular pacing for symptomatic bradycardia and cardiac resynchronisation therapy (CRT). A substantial amount of data is reported by relatively few, highly experienced centres. This study describes the first experience of LBB pacing in a high-volume device centre.

**Methods:**

Success rates (i.e. the ability to achieve LBB pacing), electrophysiological parameters and complications at implant and up to 6 months of follow-up were prospectively assessed in 100 consecutive patients referred for various pacing indications.

**Results:**

The mean age was 71 ± 11 years and 65% were male. Primary pacing indication was atrioventricular (AV) block in 40%, CRT in 42%, and sinus node dysfunction or refractory atrial fibrillation prior to AV node ablation in 9% each. Baseline left ventricular ejection fraction was < 50% in 57% of patients, mean baseline QRS duration 145 ± 34 ms. Overall LBB pacing was successful in 83 of 100 (83%) patients but tended to be lower in patients with CRT pacing indication (69%, *p* = ns). Mean left ventricular activation time (LVAT) during LBB pacing was 81 ms and paced QRS duration was 120 ± 19 ms. LBB capture threshold and R‑wave sense at implant was 0.74 ± 0.4 mV at 0.4 ms and 11.9 ± 5.9 V and remained stable at 6‑month follow-up. No complications occurred during implant or follow-up.

**Conclusion:**

LBB pacing for bradycardia pacing and resynchronisation therapy can be easily adopted by experienced implanters, with favourable success rates and safety profile.

## What’s new?

- Left bundle branch pacing is a novel pacing technique for bradycardia pacing and cardiac resynchronisation therapy.
- The majority of available data is reported by relatively few highly experienced centres.
- Left bundle branch pacing can be adopted quickly by operators without previous experience with high success and low complication rates.

## Background

For more than 60 years, permanent cardiac pacing for symptomatic bradycardia has been performed by endocardial stimulation of the right ventricle. Stimulating the right ventricle induces abnormal electrical activation and asynchronous ventricular contraction which may lead to adverse cardiac remodelling over time [1, 2]. This has been associated with an increased risk of congestive heart failure (CHF), atrial fibrillation and cardiovascular mortality [3–8]. Pacing from the right ventricular septum and pacing from the right ventricular apex are equally prone to these complications [9].

Cardiac resynchronisation therapy (CRT) improves pump function, clinical status and reduces morbidity and mortality in patients with moderate-to-severe heart failure and left bundle branch block (LBBB) [10]. The left ventricular pacing lead is positioned via the coronary sinus at the epicardium of the left ventricle. Biventricular pacing creates an electrical activation pattern that is the composite of two wave fronts originating from the right and left ventricle. It offers only modest reduction in QRS duration and left ventricular activation time since activation of the ventricles utilises non-physiological, slow cell-to-cell conduction instead of the intrinsic His-Purkinje conduction system[11–13]. Non-response to treatment approximates 30% and may partly be due to suboptimal resynchronisation [10].

Physiologic pacing is characterised by direct stimulation of the intrinsic His-Purkinje system and results in physiologic ventricular depolarisation and repolarisation. In 2017, Huang et al. first demonstrated that, by pacing beyond the region of block, left bundle branch (LBB) pacing could achieve complete correction of LBBB and improved left ventricular function in a patient with heart failure and LBBB [14]. Since then, this technique has emerged as an alternative to both traditional right ventricular pacing for bradycardia and classic CRT [15–19]. Several publications demonstrated high success rates, although the majority of scientific literature has been reported by relatively few highly experienced operators and centres [15, 20–22]. This study describes the feasibility and the safety of permanent LBBP for various pacing indications in a high-volume referral centre with no previous experience with this new pacing technique.

## Methods

### Patient selection

The first 100 consecutive patients undergoing an attempt at LBB pacing at the Catharina Hospital between January 2020 and September 2020 were prospectively investigated. Indications for pacing included bradycardia (single-chamber or dual-chamber pacemaker) or CRT (CRT pacemaker [CRT-P] or defibrillator [CRT-D]). No pre-selection of patients based on pacing indication was made. Prior to the implantation procedure the operators discussed with the patients the nonstandard but potentially more physiological nature of conduction system pacing. All procedures followed our institutional guidelines and all patients provided informed consent. Two implanting physicians participated equally in this study (i.e. L.M.R. and F.A.B.).

### Procedure

All device implantations were performed under local anaesthesia and after perioperative administration of 2 g of intravenous cefazolin. None of the patients underwent sedation or general anaesthesia. If patients were on direct oral anticoagulant therapy (DOAC), treatment was interrupted—as recommended—at least 24 h before implantation. Vitamin K antagonists were generally not interrupted, and device implantation was performed if the international normalised ratio (INR) did not exceed 3.0. Cephalic vein access for all leads using a modified Seldinger technique was the standard approach. Alternative access (i.e. axillary or subclavian vein puncture) was reserved as ‘bailout’ option.

### Left bundle branch lead implantation

**Fig. 1:**
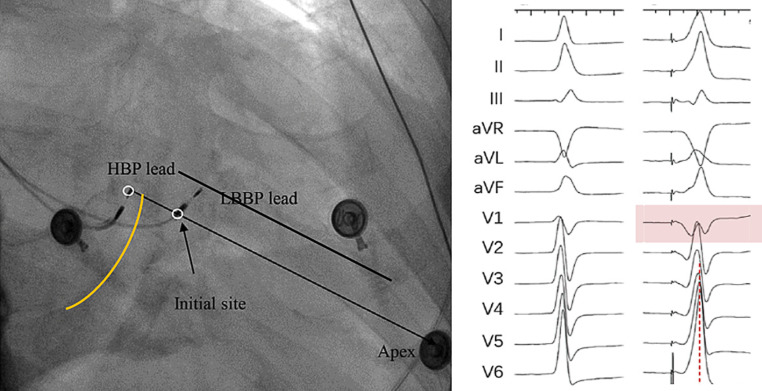
How to locate the site for left bundle branch pacing (LBBP) and electrocardiogram characteristics. Left panel: location of the His-bundle pacing (HBP) lead and LBBP lead in the right anterior oblique 30 view. Yellow line indicates location of tricuspid valve and black lines indicate demarcation of interventricular septum. Right panel: native conduction and unipolar pace mapping at the interventricular septum before lead fixation demonstrating morphology of “w” pattern with a notch at the nadir of the QRS in lead V1. Modified after Huang et al. [22] and Zhang et al. [25]

LBB pacing was performed using the SelectSecure Model 3830, 74 cm pacing lead (Medtronic Inc, Minneapolis, MN) and the C315HIS delivery sheath (Medtronic Inc, Minneapolis, MN). We based our technique on the descriptions by Huang [22]. After advancing the C315HIS sheath into the right ventricle, guided by an angled hydrophilic guidewire (Terumo Radifocus Guidewire M, 0.035″, 120 cm, Terumo Inc), the pacing lead was advanced through the sheath in right anterior oblique 20 degrees fluoroscopic projection. Unipolar pace mapping through the tip of the lead at 5V was used to scan the right ventricular septum to find the optimal pacing site, i.e. (i) a paced ECG QRS morphology in lead V1 showing a “W” morphology with a mid-notch and/or (ii) the presence of inferior lead and aVR/aVL discordance (R wave in lead II taller than in lead III, or a negative aVR and positive aVL). Fig. 1 schematically indicates finding the optimal pacing site. Using these criteria helped avoiding inadvertent lead fixation in the right ventricular outflow tract. In left anterior oblique 30 degrees fluoroscopic projection, the sheath was positioned perpendicular to the interventricular septum by anticlockwise rotation of the sheath and the pacing lead was fixated in the septum with 2 to 3 quick clockwise rotations while lead tip and sheath remained co-axial during lead fixation. Again, unipolar pacing was performed to confirm the presence of a good initial paced QRS morphology and to document the baseline pacing impedance. Subsequently, the lead was advanced by further clockwise rotations into the septum under intermittent fluoroscopic guidance. Unipolar pacing was performed after every few rotations to assess the paced QRS morphology and pacing impedance until the paced QRS morphology resembled a right bundle branch (RBB) block or RBB conduction delay pattern in V1 (QR pattern), or QS pattern with a narrow QRS. Lead depth into the interventricular myocardium was approximated by the position of the proximal radiopaque marker relative to the end of the C135HIS introducer sheath positioned against the interventricular septum. The distance between the tip of the helix and the proximal end of the radiopaque marker was approximately 1 cm. Only clockwise rotations were used as anticlockwise rotations were regarded as a risk factor for lead dislodgment, i.e. the pacing lead not being fixated by the screw and just lying in a drilled hole. We tested and recorded the left ventricular activation time (LVAT, measured as time interval from unipolar pacing spike to peak R wave at lead V5 or V6) at different outputs (usually at 1.5 V at 0.4 ms and at 5.0 V at 0.4 ms) on an electrophysiology recording system (Prucka Cardiolab, GE Healthcare, Waukesha, Wisconsin). LBB pacing was confirmed when the paced QRS morphology demonstrated either an RBB block morphology (QR or rSR’) or a QS pattern with narrow QRS (< 130 ms), and LVAT that shortened abruptly with increasing output or remained shortest and constant both at low and high outputs. Although there is no validated cut-off yet, we regarded LVAT ≤ 90 ms as an indicator of LBB capture [23]. LBB pacing was regarded unsuccessful if the abovementioned criteria could not be met.

### Endpoints

The endpoints of this study are the acute LBB pacing success rates and LBB pacing-related procedural complications within six months from implantation. The latter include interventricular septal perforation, septal coronary artery injury, transient ischaemic attack and/or stroke, lead dislodgment, device-related infection, clinically relevant decrease in R‑wave sensing, or a > 50% decrease in R‑wave sense, and an increase in LBB capture threshold > 2 V.

### Data analysis

Continuous data are presented as mean and standard deviation and discrete variables as counts and percentages, unless otherwise stated. Continuous data was compared using a Student’s t‑test. Discrete variables were analysed with chi-squared or Fisher’s exact test. A 2-sided *p*-value < 0.05 was considered statistically significant. No missing data imputation was performed. Analyses were performed using SPSS Statistics (v.25, IBM Corp., Armonk, NY).

## Results

Baseline characteristics are summarised in Tab. 1. The study population consisted of 100 consecutive patients who underwent an attempt at LBB pacing. Their mean age was 71 years and two-thirds were male. About 60% of patients had a left ventricular ejection fraction below 50%. Pacing indications included sinus node dysfunction (9%), atrioventricular (AV) block (40%), refractory atrial fibrillation prior to AV node ablation (9%), and CRT or right ventricular pacing induced heart failure (42%). The mean baseline QRS duration was 146 ms and 43% of all patients had an LBBB (Tab. 1).

**Table 1:** Baseline characteristics

Parameter	
Age	71 ± 11
*Sex*	
Male	67 (67%)
Female	33 (33%)
*Medical history*	
Hypertension	41 (41%)
Chronic kidney disease	6 (6%)
Diabetes mellitus	18 (18%)
Coronary artery disease	33 (33%)
Atrial fibrillation/flutter	40 (40%)
LV dysfunction (EF < 50%)	60 (60%)
*Pacing indication*	
Sinus node dysfunction	9 (9%)
AV block	40 (40%)
CRT or pacing-induced CMP	42 (42%)
Refractory AF and/or AVN ablation	9 (9%)
*Electrocardiogram*	
QRS width, ms	146 ± 34
Narrow QRS (< 120 ms)	21 (21%)
Wide QRS (≥ 120 ms)	79 (79%)
– LBBB	43 (54%)
– Escape rhythm, IVCD or RBBB	21 (27%)
– RV pacing	15 (19%)
*Implanted device*	
Singe-chamber pacemaker	6 (6%)
Dual-chamber pacemaker	50 (50%)
Dual-chamber defibrillator	3 (3%)
CRT‑P	9 (9%)
CRT‑D	32 (32%)

Values are *n*, (%) or mean ± standard deviation

*LV* left ventricular, *EF* ejection fraction, *AV* atrioventricular, *CRT* cardiac resynchronisation therapy, *CMP* cardiomyopathy, *AF* atrial fibrillation, *AVN* atrioventricular node, *LBBB* left bundle branch block, *RBBB* right bundle branch block, *IVCD* intraventricular conduction delay, *RV* right ventricular, *CRT‑P* cardiac resynchronisation therapy pacemaker, *CRT‑D* cardiac resynchronisation therapy defibrillator

### Success rates and implantation characteristics

In the 100 patients who had an attempt at LBB pacing, 83 were successful according to the predefined criteria. The success rate among patients referred for sinus node dysfunction, AV block or refractory atrial fibrillation prior to AV node ablation was 88% whereas in patients referred for CRT (*n* = 38) or pacing induced cardiomyopathy (*n* = 4) success rate was 69% (*p* = 0.123). The success rates between patients with and without LBBB differed significantly (67% and 88% respectively, *p* = 0.047). The overall success rate was 78% in the first and 88% in the last 50 patients (*p* = 0.183). The mean procedural time (defined as door-to-door time) decreased in the latter 50 patients from 119 ± 42 min to 104 ± 33 min (*p* = 0.111). There were no differences in baseline characteristics or in pacing indication between the first and second half of the study group. The mean LVAT was 81 ms in the 83 successful attempts. There was no difference in LVAT between patients with or without LBBB (83 ± 15 ms and 80 ± 13 ms respectively). In patients with previous right ventricular pacing there was a reduction of the mean paced QRS duration from 145 ± 34 ms to 120 ± 19 ms (*p* < 0.001). In patients with baseline LBBB, the mean reduction in QRS width after implantation was 42 ± 19 ms (from 161 ± 15 ms to 119 ± 18 ms, *p* < 0.001).

The total procedural duration was 103 ± 30 min (median 101, interquartile range (IQR) 83–118) and the fluoroscopic duration for the entire procedure was 12 ± 9 min (median 9, IQR 6–16) (Tab. 2).

**Table 2:** Implantation data (data based on successful implants)

Parameter	
LBB pacing attempted	100
*LBB pacing*	
– Successful	83
– Not successful	17
Total procedural duration, min	103 ± 30
Fluoroscopy time, min	12 ± 9
LV activation time	81 ± 14
R‑wave amplitude (mV)	12 ± 6
Unipolar threshold at 0.4 ms (V)	0.7 ± 0.4
Impedance (Ω)	739 ± 154
Baseline QRS duration	145 ± 34
Paced QRS duration	120 ± 19
Baseline QRS duration if LBBB	161 ± 15
Paced QRS duration if LBBB	119 ± 18

*LBB* left bundle branch, *LV* left ventricular, *LBBB* left bundle branch block

Tab. 3 presents follow-up data from device interrogation at 1 month and at 3 to 6 months. The mean follow-up time was 280 ± 64 days. The LBB capture threshold at implantation was 0.7 ± 0.4 V and remained stable at 1 month and 3–6 months of follow-up (0.7V ± 0.2 V and 0.8V ± 0.2 respectively). The sensed R wave at implantation was 11.9 ± 5.9 mV and increased to 14.6 ± 6.3 mV and 13.8 ± 5.4 mV at 1 and 3–6 months respectively. Impedance at implantation decreased from 739 Ω to 572 Ω and 536 Ω at 1 and 3–6 months respectively.

**Table 3:** Follow-up device data

Parameter	Baseline	1 month	3–6 months
R‑wave amplitude (mV)	11.9 ± 5.9	14.6 ± 6.3	13.8 ± 5.4
Threshold at 0.4 ms (V)	0.7 ± 0.4	0.7 ± 0.2	0.8 ± 0.2
Impedance (Ω)	736 ± 153	572 ± 82	537 ± 79

### Unsuccessful attempts

Satisfactory LBB pacing could not be obtained in 17 patients (9 male). Of thew 17 patients, 11 were referred for CRT with baseline LBBB, low ejection fraction (35 ± 11%) and enlarged left ventricular dimensions (left ventricular end-diastolic diameter 55 ± 13 mm). Six of these 11 patients had an anteroseptal myocardial infarction with local scarring on ultrasound (*p* = 0.192).

The indication in the remaining 6 failed attempts consisted of AV block in 4 patients, sinus node dysfunction and refractory atrial fibrillation prior to AV node ablation in one each.

The main reasons for failed LBB pacing included (i) the inability to position the pacing lead deep enough into the interventricular septum, mostly due to fibrosis after anteroseptal myocardial infarction, (ii) inability to obtain LVAT ≤ 90 ms due to peripheral conduction block, (iii) inability to engage the septum at the desired septal location due to lack of stable contact of the delivery sheath at the interventricular septum and (iiiv) inadequate length of the sheath to reach the desired location on the septum. The latter two reasons were noticed especially in patients with enlarged cardiac chambers. In addition, with increasing dwelling time the amount of provided support of the delivery sheath seemed to decrease.

### Complications

In the perioperative phase, there were no complications recorded, i.e. no cardiac tamponade, septal coronary artery injury, interventricular fistula or pocket haematomas.

During follow-up visits at 2 weeks, 1 month and at 3 to 6 months, no pocket infections, lead dislodgement or lead perforation occurred. In none of the patients a sudden increase in capture threshold > 1 V or loss of capture occurred. In addition, none of the patients presented with stroke/transient ischaemic attack. At the end of 6 months of follow-up there were no lead infections, lead dysfunctions or lead revisions and none of the leads had been extracted.

## Discussion

The major findings of this prospective study in 100 consecutive patients are: (i) LBB pacing is feasible in 83% of a mixed population of indications and (ii) can safely be performed without complications with (iii) satisfactory electrical lead parameters which remain stable at medium-term follow-up.

The current study also shows that this novel pacing technique is easy to perform and quickly learned by experienced device implanters. In our opinion, the learning curve is quite steep and experienced operators should be able to reach a plateau phase within 30–50 LBB pacing attempts [20].

The technique of scanning the right ventricular septum by pace mapping while looking for a “W” morphology in the V1 lead in combination with inferior lead and aVR/aVL discordance [24] was easy to perform and facilitated localisation of the optimal site for LBB pacing and avoided inadvertent lead fixation in the right ventricular outflow tract.

LBB pacing could not be performed in 17 patients (17%). Success rates were highest among patients with pacing indication for sinus node dysfunction, AV block or refractory atrial fibrillation prior to AV node ablation and lower in patients with CRT indication, an observation also reported by Padala et al. [24]. Eleven of these patients had a CRT indication. Reasons for failure included patients with extensive septal myocardial scarring as extensive fibrosis prohibited advancement of the lead with the available implant tools. Furthermore, in some cases length and/or stability of the guiding sheath we used was insufficient and made it impossible to reach the desired location on the right ventricular septum in these dilated hearts. In patients with dilated cardiomyopathy, subclavian venous puncture may be preferable over cephalic venous cutdown since the distance from venous access to the heart is larger with the latter technique. Probably the use of a longer, steerable or firmer delivery sheath could overcome these shortfalls.

LBB capture was confirmed by only two markers, i.e. (i) the paced QRS morphology in lead V1 demonstrating RBB conduction delay or block pattern, or QS pattern with narrow QRS and (ii) a stable and short LVAT. The wide spectrum of QR, rSR’, Qr, rS, and QS morphologies in lead V1 may be explained by various conduction patterns between transverse connections between the right and left bundles [25]. The LVAT was measured in precordial leads V5 or V6, whichever was longest, as a measure of the electrical activation time from the left ventricular endocardium to the epicardium of the left ventricular lateral wall. It should remain constant during both selective and non-selective LBB capture as a marker for LBB capture with fast activation propagation throughout the specialised conduction fascicules of the LBB. There are, however, no validated cut-off values of what the LVAT should be. In our cohort, the mean LVAT was 81 ms and is similar to previously reported data [16, 26, 27]. However, in patients with diffuse peripheral conduction disease, LVAT values may be prolonged, even with LBB capture. LVAT may also be extended in patients with dilated cardiomyopathy where path length to the left ventricular lateral wall is increased.

We did not include the presence of an LBB potential as criterion for LBB capture. Previous studies reported a high variability in recording these potentials, from 28% to 80% of cases [21, 28, 29]. In patients with LBBB, LBB potentials can only be recorded during restoration of left bundle conduction, e.g. by temporary His-bundle pacing [27]. In addition, Padala et al. demonstrated in a recent series of 305 cases that an LBB potential could be found in only 41% of patients with successful LBB lead implantation [24]. In their study, only 11% of patients had baseline LBBB. In our study just over 40% of patients had an LBBB. It may be questionable whether documenting an LBB potential is a prerequisite of confirmation of LBB capture [28, 30]. At the optimal pacing sites, LBB capture threshold is almost invariably lower than local myocardial capture and could be helpful in differentiating selective versus non-selective LBB pacing. The resulting difference in QRS morphology during unipolar pacing is also an indication for LBB capture as changing pacing output would not change the QRS morphology with myocardial only stimulation. Since we did not record LBB potentials, determination of selective or non-selective LBB pacing (i.e. direct activation of both LBB and local myocardium) was not possible. However, Su et al. recently published that in 460 patients with selective LBB capture at implant, at follow-up 191 patients still had selective LBB capture whereas 292 patients had non-selective LBB capture, which is probably caused by a decrease in septal myocardial capture threshold in the post-operative phase [20].

The short-term pacing and sensing parameters were excellent and compared favourable to traditional right ventricular pacing. During follow-up, the lead parameters remained very stable. Probably lead maturation may benefit from a deep intramyocardial lead position when compared to traditional endocardial leads.

In this study neither implantation related nor lead performance or device related complications were recorded during the entire follow-up. Importantly, results should be interpreted with caution since sample size was relatively small and follow-up short.

### Unknowns and future perspectives

Mechanical effects of the contracting myocardium on the deep-seated lead body may affect lead longevity and needs to be further studied. In addition, the ability to safely extract these leads in the future should be investigated [14].

Although LBB pacing seems a potential alternative to conventional CRT in patients with LBBB, it is still unknown which patients will benefit most from this novel technique. Upadhyay et al. showed that LBBB with intact Purkinje activation was present in approximately only 36% patients in their study [31]. The presence of conduction delay distally from the pacing site may attenuate effective resynchronisation. However, regions of conduction delay or block may also influence the effectiveness of coronary sinus left ventricular pacing. In contrast, if the LBBB can be corrected by LBB pacing, the region of block is most likely situated proximally of the pacing site and with intact distal Purkinje conduction [31]. Indeed, in patients with successful LBB pacing mean LVAT values were similar when comparing baseline LBBB and non-LBBB (83 ms versus 81 ms respectively).

The predictive value of demonstrating scar tissue in the septal region of the LBB on successful LBB pacing should also be investigated as this could prevent futile attempts at LBB pacing, especially in patients with ischaemic cardiomyopathy.

The current delivery sheath and lead were not specifically developed for LBB pacing and new developments in delivery sheath and possibly lead design may improve the success rate. In addition, the use of stylet-driven leads may further increase the success rate of LBB pacing. Concerns about the use of these leads include their larger diameter in comparison to the SelectSecure Model 3830 lead used in this study. Larger leads may carry additional risk of perforation of septal coronary artery branches. In addition, extendable screws lack the isodiametric shape at the location where the helix exits the helix case, which might limit lead penetration into the septum. Long-term data on lead behaviour in deep septal position and lead performance are still lacking for stylet-driven leads.

Finally, large prospective randomised studies are needed to further confirm the feasibility, long-term safety and usefulness of LBB pacing for various conditions.

## Conclusions

This prospective single-centre study demonstrated that LBB pacing for bradycardia pacing and resynchronisation therapy can be adopted quickly by a centre without previous experience with this technique with favourable success rates and safety profile.
